# Effect of *in ovo* feeding of γ-aminobutyric acid combined with embryonic thermal manipulation on hatchability, growth, and hepatic gene expression in broilers

**DOI:** 10.5713/ab.22.0099

**Published:** 2022-06-30

**Authors:** Chris Major Ncho, Akshat Goel, Vaishali Gupta, Chae-Mi Jeong, Yang-Ho Choi

**Affiliations:** 1 Department of Animal Science, Gyeongsang National University, Jinju 52828, Korea; 2 Institute of Agriculture and Life Sciences, Gyeongsang National University, Jinju 52828, Korea; 3 Division of Applied Life Sciences (BK21 Plus Program), Gyeongsang National University, Jinju 52828, Korea

**Keywords:** Antioxidant Status, Broiler, γ-Aminobutyric acid (GABA), *In ovo* Feeding, Thermal Manipulation

## Abstract

**Objective:**

This study investigated the effects of *in ovo* feeding of γ-aminobutyric acid (GABA) and embryonic thermal manipulation (ETM) on growth performance, organ indices, plasma biochemical parameters, hepatic antioxidant levels, and expression of lipid metabolism-related genes in broilers.

**Methods:**

Two hundred and fifty eggs were assigned to one of four treatments: control eggs incubated under standard conditions (CON); eggs that received an *in ovo* injection of 10% GABA on day 17.5 of incubation (G10); thermally manipulated eggs between days 10 and 18 of incubation at 39.6°C for 6 h daily (TM); and eggs that received both treatments during incubation (G10+TM). After 28 days of rearing, five birds per treatment were selected for blood and organ sampling.

**Results:**

No differences were found in hatchability or growth parameters among different treatment groups. Hepatic gene expression of catalase (*CAT*) and glutathione peroxidase 1 (*GPx1*) was upregulated (p = 0.046 and p = 0.006, respectively) in the G10+TM group, while that of nuclear factor erythroid 2–related factor 2 (*NRF2*) was upregulated (p = 0.039) in the G10 group. In addition, the relative gene expression of NADPH oxidase 1 (*NOX1*) was significantly lower (p = 0.007) in all treatment groups than that in the CON group. Hepatic fatty acid synthase (*FAS*) levels and average daily feed intake (ADFI) of last week showed a positive correlation (r = 0.50, p = 0.038). In contrast, the relative gene expression of the extracellular fatty acid-binding protein (*EXFAB*) and peroxisome proliferator-activated receptor-γ (*PPAR-γ*) were positively correlated (r = 0.48, p = 0.042 and r = 0.50, p = 0.031) with the overall ADFI of birds.

**Conclusion:**

Taken together, the results of this study suggest that the combination of *in ovo* feeding of GABA and ETM can enhance hepatic antioxidant function in broilers.

## INTRODUCTION

The poultry production industry faces several challenges that negatively affect birds: stocking density, disease infection, and heat stress (HS). Recent literature indicates that these stressors are often associated with the overproduction of reactive oxygen species (ROS), leading to oxidative stress [1,2]. Birds have developed various antioxidant defense systems to adapt to and survive in the environment. Indeed, in avian species, cell signaling and stress adaptation are maintained by the redox status [3]. The redox balance can be regulated by various systems. The first step is to lower the action of enzymes such as NADPH oxidase (NOX), which is responsible for ROS production, thus limiting the production of free radicals by decreasing oxygen accessibility. The combined action of three antioxidant enzymes ensures another line of defense: catalase (CAT), superoxide dismutase (SOD), and glutathione peroxidase (GPx) [4]. SOD catalyzes the dismutation of O_2_^−^ into O_2_ and H_2_O_2_. Furthermore, GPx and CAT catalyze the breakdown of H_2_O_2_ into H_2_O and oxygen [5]. These defense systems are triggered by the activation of nuclear factor (erythroid-derived 2)-like 2 (NRF2), which is responsible for the expression of antioxidant and detoxification-related genes [6].

Several strategies have been proposed to alleviate oxidative stress in broiler chickens. One approach was dietary supplementation with γ-aminobutyric acid (GABA) [7]. GABA is a neurotransmitter that regulates vital functions such as blood pressure and respiration [8]. Supplementation with GABA is particularly effective against heat-induced oxidative stress [9]. To mitigate HS, previous studies have used GABA as a feed additive for broilers [10] or provided it to the drinking water of laying hens [11]. Furthermore, our previous study showed that *in ovo* GABA feeding can improve the antioxidant status in chicks exposed to acute HS [12].

Another approach against heat-induced oxidative stress is epigenetic temperature adaptation. This method can be applied during pre- or early- post-hatch ontogenesis to modulate gene expression and provide long-lasting adaptation to the environment [13]. The most recent of these techniques is embryonic thermal manipulation (ETM). Furthermore, studies have demonstrated that a particular pattern of incubation temperature can affect the metabolic rate of hatchlings. Indeed, the results showed that ETM improves defense mechanisms by enhancing the antioxidant and immune responses of broilers [14].

In the present study, we hypothesized that *in ovo* feeding of GABA combined with ETM could modulate post-hatch gene expression in broilers. Thus, we evaluated the growth performance, organ indices, plasma biochemical parameters, and hepatic expression of genes related to antioxidant defenses and lipid metabolism in broilers.

## MATERIALS AND METHODS

All experimental procedures were approved by the Institutional Animal Care and Use Committee of Gyeongsang National University (GNU-200916-C0058).

### Incubation, *in ovo* feeding procedure and feeding trial

Two hundred and fifty hatching eggs were obtained from 37-week-old Indian River breeder hens housed at a local breeder farm (Hapcheon, Korea). Following standard incubation conditions, the eggs were incubated in an incubator (Rcom Co., Ltd., Gimhae, Korea). Briefly, from embryonic day (ED) 1 to 18, eggs were subjected to 37.8°C and 56% relative humidity (RH), and from ED 18 until hatching, the incubation temperature was maintained at 36.8°C with 70% RH. On ED 10, after candling, non-fertilized eggs were removed from the incubator. The eggs were distributed in four groups of equal numbers (n = 48) with similar weights (61.0± 2 g) using the Solver module of Microsoft Excel (Microsoft Excel 2016; Microsoft Corp., Washington, USA). Each group consisted of eight replicates with six eggs. In our previous study, we found no significant differences (hatching and biological parameters) between the sham control (distilled water injected) and the non-injected control; therefore, we did not include a sham treatment in this trial. The groups were the followings: i) control eggs without *in ovo* injection and incubated at standard temperature (CON); ii) eggs injected at 17.5 days of incubation with 0.6 mL of 10% (0.1 g/mL) GABA (#A2129; Sigma-Aldrich Inc., St. Louis, CA, USA) dissolved in distilled water (G10); iii) thermally manipulated eggs exposed to 39.6°C for 6 h (from 10:00 h to 16:00) daily from ED 10 to 18 (TM); and iv) eggs that received both previous treatments during incubation (G10+TM).

The solution and methodology used for the *in ovo* feeding procedure were the same as those previously described [15,16]. Briefly, a hole was drilled from the blunt end of the eggs and subsequently injected with a 1 mL syringe with a 23 G and 1-inch needle (KOVAX-SYRINGE; Korea Vaccine Co., Ltd. Seoul, Korea). Therefore, the injection targeted the amnion with a depth of approximately 2.5 cm.

Immediately after the injection, eggs were sealed using surgical tape (3M Micropore, Saint Paul, MN, USA) and incubated. The control eggs were removed from the incubator for the same period but returned without injection. After hatching, one-day-old broilers (n = 140) with similar weights were selected to equalize the starting weight between the cages and treatments. The birds were raised in battery brooders in a thermally controlled environment at 34°C±1°C and 50% RH for one-day-old broilers, and then the temperature was gradually decreased to 22°C±1°C on day 28. Commercial feed (Nonghuyp Feed Inc., Seoul, Korea) (Table 1) and water were provided *ad libitum* under continuous lighting. Each treatment consisted of five cages with seven chicks each.

### Blood, tissue sampling, and plasma biochemical parameters analysis

On day 28, one bird from each cage was randomly selected for blood and tissue sampling. The birds were euthanized with carbon dioxide before sampling. Blood was collected by heart puncture and transferred into heparinized vacuum containers (#367874; BD Co., Ltd., Franklin Lakes, NJ, USA). The blood samples were then centrifuged at 2,000×*g* for 10 min at 4°C, and plasma was collected and stored at −20°C for later analysis. Tissues were sampled and weighed (liver, spleen, bursa, and heart), and the liver samples were snap-frozen in liquid nitrogen and stored at −80°C for further analysis. Plasma metabolite concentrations were measured using a VetTest Chemistry Analyzer (IDEXX Co., Ltd., Westbrook, CT, USA) with dry-slide technology, following the manufacturer’s instructions.

### Real-time polymerase chain reaction for mRNA quantification

Following the manufacturer’s protocol, total RNA was extracted from the liver using TRIzol reagent (Thermo Fisher Scientific, Waltham, MA, USA). The concentrations and purities of the samples were determined by measuring the optical density of each sample using a NanoDrop spectrophotometer (Thermo Fisher Scientific, USA). Subsequently, reverse transcription was conducted using the PrimeScript first-strand cDNA synthesis kit (Takara, Tokyo, Japan) following the manufacturer’s instructions. The cDNA was then used for performing real-time polymerase chain reaction (PCR) using the StepOnePlus real-time PCR system (Life Technologies, Carlsbad, CA, USA) according to the following protocol: 10 min at 95°C, followed by 40 cycles of 15 s at 95°C and 1 min at 60°C. Each reaction well was composed of a 20 μL volume containing Power SYBR Green PCR master mix (Life Technologies, USA) and 10 pmol concentration of forward and reverse primers specific for each gene and cDNA. Table 2 lists the primer sequences used in this study. Gene quantification was normalized to the Ct values of glyceraldehyde-3-phosphate dehydrogenase (*GAPDH*) and *β-actin*. Relative expression was determined using the 2^−ΔΔct^ algorithm. The geometric means of both references were used to calculate the expression levels of the target genes.

### Statistical analysis

Before the analysis, all assumptions related to the application of parametric tests were assessed. Data were analyzed via one-way analysis of variance (ANOVA) using the “general linear model procedure” in the SAS software (version 9.4; SAS Institute Inc., 2009). A Tukey’s post hoc test was performed following a significant p-value to assess the differences among means. Concerning hepatic gene expression, orthogonal planned contrasts were made to evaluate specific comparisons using the “Contrast statement” of the SAS software [17]. Differences were considered statistically significant at p≤0.05, and p≤0.1 was considered a trend. Moreover, gene expression patterns were detected by hierarchical clustering [18] using the package “ComplexHeatmap” in R software version 4.0.3 (R Core Team, 2020). Finally, Pearson’s correlation analysis was performed for the evaluated parameters to develop a correlation matrix using the “CORR procedure” in the SAS software.

## RESULTS

### Hatching parameters, growth performance, and organ indexes

No significant differences were observed in the hatching parameters (Table 3) among different treatment groups. *In ovo* feeding of GABA and ETM did not significantly affect hatchability or the average hatchling weight.

Body weight, average daily weight gain (ADWG), and aver age daily feed intake (ADFI) were similar in all treatment groups regardless of the period. However, the G10+TM group had an overall higher (p = 0.018) feed conversion ratio (FCR) than the CON group from days 8 to 21 of the post-hatch period (Table 4).

The organ index results are presented in Table 5. The ab solute spleen, bursa, and heart weights did not differ across different treatment groups. In contrast, absolute liver weight was statistically different (p = 0.046) among the treatment groups, with the highest value recorded in the G10 group and the lowest in the G10+TM group. Significant differences were observed in the relative weight of the bursa among the treatment groups. Chicks in the G10+TM group had a higher (p = 0.008) relative bursa weight than those in the CON group did. Finally, the relative liver, spleen, and heart weights were similar among all the treatment groups.

### Plasma biochemical parameters

Table 6 presents the effects of *in ovo* feeding with GABA and thermal manipulation on the plasma biochemical parameters of broilers. Plasma glucose, triglyceride, and cholesterol levels were not affected by any treatment. However, plasma total protein levels were higher (p = 0.019) in chicks in the G10+TM group than those in the CON group.

### Hepatic mRNA relative expression

Tables 7 and 8 show the results of the ANOVA procedure and contrast analysis performed on the hepatic mRNA relative expression of the genes studied. Significant differences among the treatment groups were revealed for the expression of antioxidant-related genes. The glutathione peroxidase 1 (*GPx1*) gene was significantly upregulated (p = 0.039) in chicks in the G10+TM group compared to those in the CON group. In contrast, significantly lower (p = 0.039) expression of the NADPH oxidase 1 (*NOX1*) gene was observed in the G10 and G10+TM treatment groups than in the CON treatment group. Furthermore, the contrast analysis results showed that the chicks from the G10+TM group exhibited higher (p = 0.046) *CAT* gene expression than the CON group chicks. Although not statistically significant, *NOX4* gene expression showed a decreasing tendency (p = 0.077) in the G10 group. In addition, orthogonal contrast analysis showed that the *NRF2* gene was significantly upregulated (p = 0.039) in birds fed GABA *in ovo* (G10). Although a numerically lower relative expression of the lipid metabolism gene in the G10+TM group was continuously observed, neither the ANOVA procedure nor the contrast analysis revealed significant differences among all groups.

**Figure 1:**
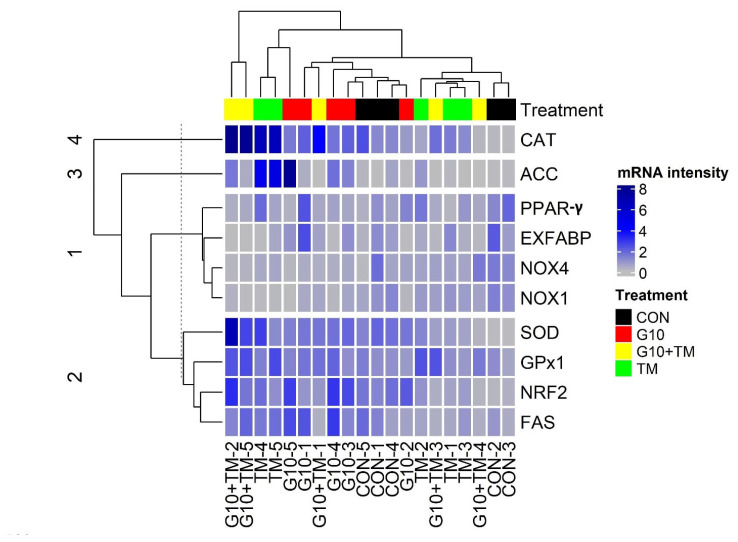
Hierarchical cluster tree of hepatic gene expression. The genes panel included antioxidant genes (*CAT*, *SOD*, *GPx1*, *NOX1*, *NOX4*, and *NRF2*) and lipid metabolism genes (*ACC*, *FAS*, *EXFABP*, and *PPAR-γ*). Each row represents a gene and each column represents an experimental unit belonging to a specific treatment. The treatments are described as follows: CON, control eggs without *in ovo* injection and incubated at standard temperature; G10, eggs injected at 17.5 days of incubation with 0.6 mL of 10% γ-aminobutyric acid dissolved in distilled water; TM, thermally manipulated eggs exposed to 39.6°C for 6 h daily from embryonic day 10 to 18; G10+TM, eggs that received both previous treatments during incubation. Gene expression analysis was performed using real-time quantitative polymerase chain reaction (RT-qPCR) with *GAPDH* and *β-actin* as reference genes. The tree was constructed using the package “ComplexHeatmap” of the R software version 4.0.3 (R Core Team, 2020). *CAT*, catalase; *ACC*, acetyl-CoA carboxylase; *PPAR-γ*, peroxisome proliferator-activated receptor-gamma; *EXFABP*, extracellular fatty acid-binding protein; *NOX4*, nicotinamide adenine dinucleotide phosphate oxidase 4; *NOX1*, nicotinamide adenine dinucleotide phosphate oxidase 1; *SOD*, superoxide dismutase; *GPx1*, glutathione peroxidase 1; *NRF2*, nuclear factor erythroid 2-related factor 2; *FAS*, fatty acid synthase.

Gene expression data were clustered into four groups based on Euclidean distances (Figure 1). Two large clusters were identified: the first was composed of *NOX1*, *NOX4*, extracellular fatty acid-binding protein (*EXFABP*), and peroxisome proliferator-activated receptor-γ (*PPAR-γ*), with *NOX1*, *NOX4*, and *EXFABP* being downregulated (bright gray bars), whereas *PPAR-γ* expression was decreased or unchanged compared to the control. The second-largest cluster included *SOD*, *GPx1*, *NRF2*, and fatty acid synthase (*FAS*) genes, which were moderately upregulated (blue bars). Finally, *CAT* and acetyl-CoA carboxylase (*ACC*) were the only genes in separate clusters. *CAT* was robustly upregulated (dark blue bars) in the treatment groups relative to the control group.

**Figure 2:**
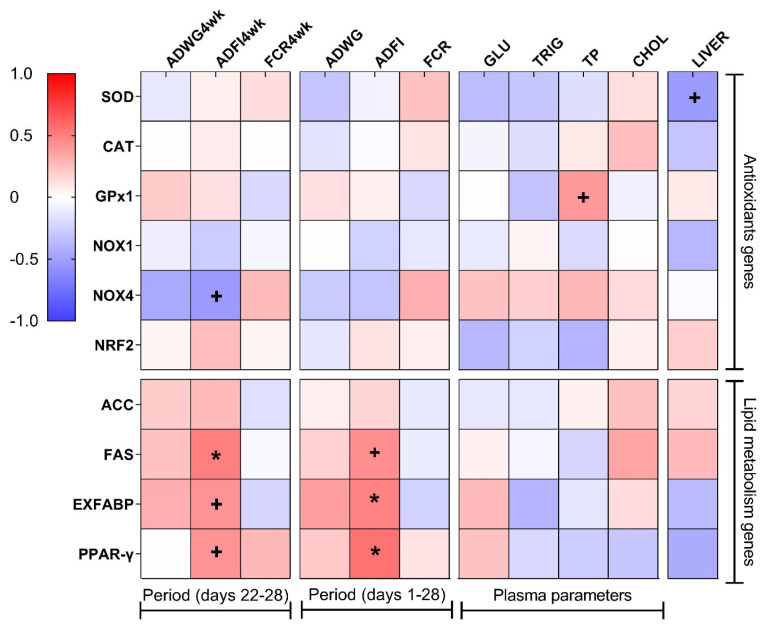
Pearson correlation heat map between the relative mRNA levels of the genes studied, the growth parameters, the plasma biochemical parameters, and the relative liver weight in broiler chickens. The red color indicates a positive correlation, the blue color indicates a negative correlation and the white color indicates no correlation. Pearson r values were calculated using the “CORR procedure” of the SAS software version 9.4 (SAS Institute Inc., 2009). ADWG4wk, average daily weight gain of the last week; ADFI4wk, average daily feed intake of the last week; FCR4wk, feed conversion ratio of the last week; ADWG, average daily weight gain; ADFI, average daily feed intake; FCR, feed conversion ratio; GLU, glucose; TRIG, triglycerides; TP, total protein; CHOL, cholesterol; LIVER, relative liver weight; *SOD*, superoxide dismutase; *CAT*, catalase; *GPx1*, glutathione peroxidase 1; *NOX1*, nicotinamide adenine dinucleotide phosphate oxidase 1; *NOX4*, nicotinamide adenine dinucleotide phosphate oxidase 4; *NRF2*, nuclear factor erythroid 2-related factor 2; *ACC*, acetyl-CoA carboxylase; *FAS*, fatty acid synthase; *EXFABP*, extracellular fatty acid-binding protein; *PPAR-γ*, peroxisome proliferator-activated receptor-gamma. + Indicates a trend at the 0.1 level. * Correlation is significant at p<0.05 level.

Figure 2 presents the results of Pearson’s correlation anal ysis between relative gene expression, growth parameters, plasma biochemical parameters, and relative liver weight. The overall ADFI was positively correlated (red color) with both relative *EXFABP* (r = 0.48, p = 0.042) and *PPAR-γ* (r = 0.55, p = 0.031) gene expression. Interestingly, the same positive correlation was observed between ADFI during the final week of the trial (ADFI4wk) and *FAS* (r = 0.50, p = 0.038) gene expression.

## DISCUSSION

Modern broilers have undergone decades of intensive genetic selection for rapid growth and muscle yield, rendering them particularly susceptible to oxidative stress [19]. Oxidative stress occurs when there is an overproduction of free radicals, such that the antioxidant system can no longer act as a neutralizer [20]. There are numerous options for combating oxidative stress in poultry. The first step is to limit ROS production by reducing the activity of NOX enzymes. The NOX protein complex mainly transfers electrons from NADPH to oxygen molecules, resulting in O_2_^−^ production [21]. The second is to increase the scavenging of free radicals through antioxidant defenses. In chickens, the first line of antioxidant defense consists of SOD, CAT, and GPx [22]. Activation of the *NRF2* gene is a critical step in the antioxidant modulation pathway [6]. NRF2 is involved in the synthesis of the three major antioxidant enzymes [1]. This study evaluated the hepatic gene expression of *CAT*, *SOD*, *GPx1*, and *NRF2* after ETM and *in ovo* feeding with GABA. Only birds in the G10 group had significantly higher hepatic gene expression of NRF2 than the CON group. Both *GPx1* and *CAT* were significantly upregulated only in the G10+TM group (Table 8).

Interestingly, *SOD*, *CAT*, and *GPx1* were upregulated in all treatment groups, with the G10+TM group always showing the highest expression (Table 7). Furthermore, all three treatments downregulated the expression of *NOX1*. Our previous study highlighted that *in ovo* feeding of GABA increased the plasma total antioxidant activity in heat-stressed chicks [12]. Others have also reported enhanced GPx activity but reduced oxidation levels in birds fed GABA during stress [23]. The ability of GABA to mitigate oxidative stress is linked to its ability to promote glutamate levels [24], thus promoting the synthesis of antioxidant enzymes, such as GPx [25]. However, the mechanism by which GABA could modulate NRF2 remains unclear and requires further research. Additionally, ETM ameliorated heat-induced oxidative stress by downregulating the hepatic *NOX* gene family [26]. Another study evaluating the effects of cold-induced oxidative stress in broilers also revealed that ETM could lower the expression of *NOX4* in the liver, spleen, and heart [27]. Based on the concept of an antioxidant system integrated into cells, we hypothesized that a combination of antioxidants would be more effective than a single antioxidant [22]. Therefore, these findings indicate the combined effect of *in ovo* feeding and thermal manipulation on the enhancement of hepatic antioxidant capacity in broilers.

Although no significant results were found across differ ent treatment groups regarding lipid metabolism-related genes, we found correlations among hepatic gene expression of *FAS*, *EXFABP*, *PPAR-γ*, and ADFI (Figure 2). *FAS* and *ACC* are two important genes that encode key enzymes in *de novo* fatty acid synthesis in birds [28]. FAS utilizes malonyl-CoA, catalyzed by ACC, to produce long-chain fatty acids. Similar to our results, *FAS* expression increased with increased feed intake in chickens [29]. This might explain why the correlation was stronger between ADFI in the last phase of the trial because chicks increased their feed intake with age. PPAR-γ is a transcription factor involved in the regulation of adipogenesis [30]. EXFABP belongs to a family of fatty acid-binding proteins that mediate the metabolism and transportation of lipids. The positive correlation between these genes and the overall ADFI in this study may indicate higher lipid deposition in birds with the highest feed consumption.

Hierarchical clustering revealed that the gene panel could be separated into four clusters, with two larger clusters. The first cluster included a set of downregulated genes (*NOX1*, *NOX4*, *EXFABP*, and *PPAR-γ*). Since NOXs family genes are responsible for ROS production, we can hypothesize that limited lipid deposition is associated with reduced ROS production. The second largest cluster included the *SOD*, *GPx1*, *NRF2*, and *FAS* genes, which were predominantly upregulated in our treatment groups. The first line of antioxidant enzymes showed increased gene expression in G10, TM, and G10+TM groups. The synthesis of this array of protective molecules results from the activation of the NRF2 transcription factor to maximize antioxidant protection and maintain internal redox balance [20]. This might explain why these genes were in the same cluster. In addition, the higher feed intake recorded in both G10 and TM during the final phase of our trial accounted for the upregulation of *FAS*.

This study aimed to not only assess the effects of *in ovo* feeding of GABA and ETM in broilers, but also to elucidate their potential potentiating effects when treated together. Because all treatments were applied during incubation, the primary outcomes to be measured were the hatching parameters. No significant differences were observed in hatchability or hatchling weight across all the treatment groups. Previous results have shown that *in ovo* feeding of amino acids [31], especially GABA, does not reduce hatchability in broilers. This result indicates that a high intake of exogenous GABA during incubation is not detrimental to chicken embryos, and thus, *in ovo* feeding of GABA is safe and can be useful for the poultry industry.

Our results also highlight that the ETM used in this study did not reduce hatchability. On the other hand, the effect of ETM on hatchability is inconsistent in the literature, reporting a significant reduction [32], increase, or no effect [16]. The discrepancies in the results may be attributed to the duration and intensity of thermal manipulation. Indeed, some studies [33] have reported decreased growth and hatchability in embryos exposed to continuous ETM. Therefore, intermittent ETM may be more appropriate to avoid a drastic reduction in hatchability. Finally, the combination of *in ovo* feeding of GABA and ETM, which does not significantly affect hatchability, indicates that both techniques can be implemented in this configuration without harming the embryo.

Post-hatch growth performance mostly did not show any differences between groups; only FCR during the 8 to 21 days period was increased in the G10+TM group. Studies evaluating *in ovo* feeding have consistently reported its significant effects on growth within the first two weeks of the rearing period. Growth performance has been reported to improve after *in ovo* feeding with various amino acids [34]. Although ADWG and FI were higher in the G10 group, no significant differences were found in our trial. Differences in nutrients, concentrations, and dosages may explain these results. Although the literature reports the merits of ETM, its beneficial effects on growth parameters have not been recognized. Likewise, ETM does not affect or reduce the growth performance [35]. Thus, the higher FCR recorded in the G10+TM group may be associated with thermal manipulation rather than *in ovo* feeding, because the G10 group consistently performed better.

The organ index is an important indicator that reflects the developmental status of organs. The immune organ index is especially significant because it often correlates with the magnitude of immune function [36]. In this study, birds in the G10+TM group had the highest relative bursa weights. Similarly, Tang and Chen [37] found an increased bursa index and improved immune function in broilers supplemented with GABA at 14 and 21 days of rearing. Evidence suggests that GABA plays a role in immunity by modulating cytokine secretion and modifying immune cell secretion [38]. However, none of the trials evaluating the potential effects of GABA on the bursa have conducted an in-depth study to elucidate the mechanism underlying its action. Thus, although GABA effectively enhances the bursa index, the explanations on this topic remain speculative, and further research is needed. In addition, the immunity-promoting abilities of ETM in poultry have already been demonstrated [26]. Thus, these results suggest the concomitant action of *in ovo* feeding of both GABA and ETM on the immune status of broilers.

In conclusion, the results of this study showed that *in ovo* GABA feeding and ETM increased the bursa index and improved hepatic antioxidant gene expression in broilers when treated together during incubation. Therefore, further investigation of the potential synergistic effect of the two methods for alleviating heat-induced oxidative stress in broilers will be of great interest.

## Figures and Tables

**Table 1:** Feed composition of the experimental diets from 0 to 28 days of age^1)^

Items	Starter (0 to 7 d)	Grower (8 to 21 d)	Finisher (22 to 28 d)
Ingredients (%)			
Corn grain	38.97	45.87	39.68
Wheat grain	15.00	15.00	25.00
Soybean meal (42.6% CP)	32.00	25.60	20.60
Corn gluten	3.00	2.64	3.00
Meat and bone meal	2.00	2.00	2.50
Animal fat	4.00	3.88	4.54
Salt	0.25	0.25	0.25
Tricalcium phosphate	1.30	1.04	0.86
Limestone	1.26	1.22	1.26
Sodium bicarbonate	0.00	0.02	0.00
L-threonine	0.12	0.16	0.16
Lysine	1.23	1.43	1.32
DL-methionine	0.33	0.34	0.29
Choline chloride (50.0%)	0.03	0.03	0.03
Premix^2)^	0.20	0.20	0.20
Phytase	0.05	0.05	0.05
Anti-coccidia	0.01	0.01	0.01
Calculated nutrients (%)			
Crude protein	23.00	20.50	19.50
Crude fat	6.31	6.36	6.90
Crude fiber	3.01	2.80	2.68
Crude ash	5.99	5.34	5.02
Calcium	1.01	0.90	0.86
Available phosphorus	0.60	0.53	0.49
Digestible lysine	1.43	1.24	1.09
Digestible methionine+cystine	1.07	0.95	0.86
Copper (ppm)	82.21	81.04	80.78
Zinc (ppm)	100.27	96.63	97.33
Metabolizable energy (kcal/kg)	3,050	3,150	3,200

1) Nonghuyp Feed Inc. (Seoul, Korea).

2) Trace minerals and vitamins provided per kilogram of premix: vitamin A, 12,000,000 IU; vitamin D_3_, 3,000,000 IU; vitamin E, 40,000 IU; vitamin K_3_, 2,000 IU; vitamin B_1_, 2,000 mg; vitamin B_2_, 5,000 mg; vitamin B_6_, 3,000 mg; vitamin B_12_, 20 mg; niacin, 40,000 mg; pantothenic acid, 10,000 mg; folic acid, 1,000 mg, iron, 88,000 mg; copper, 72,600 mg; zinc, 60,000 mg; manganese, 66,000 mg; iodine, 990 mg; selenium, 220 mg; cobalt, 330 mg.

**Table 2:** Oligonucleotide primer sequences for RT-qPCR

Gene	Sequence	Accession number
*ACC*	F: CACTTCGAGGCGAAAAAC R: GGAGCAAATCCATGACCA	XM_015295697.2
*CAT*	F: ACCAAGTACTGCAAGGCGAA R: TGAGGGTTCCTCTTCTGGCT	NM_001031215.1
*EXFABP*	F: GGAGGACCTTGCACATGA R: GTGTAGTTCCGCTCCCTA	NM_205422.1
*FAS*	F: CAATGGACTTCATGCCTC R: GCTGGGTACTGGAAGACA	NM_205155.3
*GPx1*	F: AACCAATTCGGGCACCAG R: CCGTTCACCTCGCACTTCTC	NM_001277853.2
*NOX1*	F: GCGAAGACGTGTTCCTGTAT R: GAACCTGTACCAGATGGACTTC	NM_001101830.1
*NOX4*	F: CCTCTGTGCTTGTACTGTGTAG R: GACATTGGAGGGATGGCTTAT	NM_001101829.1
*NRF2*	F: CAGAAGCTTTCCCGTTCATAGA R: GACATTGGAGGGATGGCTTAT	NM_205117
*PPAR-γ*	F: TCAGGTTTGGGCGAATGC R: CGCTCGCAGATCAGCAGA	XM_040646063.1
*SOD*	F: AGGGGGTCATCCACTTCC R: CCCATTTGTGTTGTCTCCAA	NM_205064.1
*β-actin*	F: ACCGGACTGTTACCAACA R: GACTGCTGCTGACACCTT	NM_205518.1
*GAPDH*	F: TTGGCATTGTGGAGGGTCTTA R: GTGGACGCTGGGATGATGTT	NM_204305.1

RT-qPCR, real-time quantitative polymerase chain reaction; *ACC*, acetyl-CoA carboxylase; *CAT*, catalase; *EXFABP*, extracellular fatty acid-binding protein; *FAS*, fatty acid synthase; *GPx1*, glutathione peroxidase 1; *NOX1*, nicotinamide adenine dinucleotide phosphate oxidase 1; *NOX4*, nicotinamide adenine dinucleotide phosphate oxidase 4; *NRF2*, nuclear factor erythroid 2-related factor 2; *PPAR-γ*, peroxisome proliferator-activated receptor gamma; *SOD*, superoxide dismutase; *GAPDH*, glyceraldehyde-3-phosphate dehydrogenase.

**Table 3:** Effects of *in ovo* feeding of γ-aminobutyric acid and embryonic thermal manipulation on hatchability parameters

Parameters	Treatments^1)^				SEM	p-value

	CON	G10	TM	G10+TM		
Number of fertile eggs	48	48	48	48	NA	NA
Number of hatchlings	46	43	45	41	NA	NA
Hatchability (%)	95.8	89.6	93.7	85.4	1.9	0.255
Hatchling weight (g)	40.9	40.4	39.7	40.0	0.23	0.296

Hatchability was calculated based on n = 8 repetitions per treatment.

SEM, standard error of the mean; NA, not applicable.

1) The treatments are described as follows: CON, control eggs without *in ovo* injection and incubated at standard temperature; G10, eggs injected at 17.5 days of incubation with 0.6 mL of 10% γ-aminobutyric acid dissolved in distilled water; TM, thermally manipulated eggs exposed to 39.6°C for 6 h daily from embryonic day 10 to 18; G10+TM, eggs that received both previous treatments during incubation.

**Table 4:** Effects of *in ovo* feeding of γ-aminobutyric acid and embryonic thermal manipulation on growth, feed intake, and feed conversion ratio in broiler chickens

Parameters	Treatment^1)^				SEM	p-value

	CON	G10	TM	G10+TM		
BW (g)						
Day old	40.3	40.2	39.9	40.0	0.07	0.149
7 d	155.1	154.3	152.2	167	0.10	0.161
21 d	909.1	901.7	879.8	881.7	8.10	0.528
28 d	1,450.2	1,493.8	1,431.3	1,419	18.3	0.526
ADWG (g)						
1 to 7 d	16.4	16.2	16.1	18.2	0.37	0.151
8 to 21 d	53.8	53.4	52	51.1	0.52	0.211
22 to 28 d	77.3	84.6	78.8	76.8	2.31	0.649
1 to 28 d	49.1	51.4	48.9	48.6	1.40	0.628
ADFI (g)						
1 to 7 d	15.4	14.8	14.6	16.9	0.35	0.084
8 to 21 d	67.6	69.1	66.1	67.3	0.58	0.337
22 to 28 d	116	125.8	119.9	112.1	1.96	0.067
1 to 28 d	66.3	69.9	66.8	65.4	1.20	0.095
FCR (g/g)						
1 to 7 d	0.94	0.91	0.91	0.92	0.01	0.585
8 to 21 d	1.25^b^	1.28^ab^	1.26^ab^	1.30^a^	0.02	0.018
22 to 28 d	1.51	1.49	1.52	1.51	0.03	0.984
1 to 28 d	1.23	1.23	1.23	1.25	0.02	0.873

Values were obtained from n = 5 repetitions per treatment.

SEM, standard error of the mean; BW, body weight; ADWG, average daily weight gain; ADFI, average daily feed intake; FCR, feed conversion ratio.

1) The treatments are described as follows: CON, control eggs without *in ovo* injection and incubated at standard temperature; G10, eggs injected at 17.5 days of incubation with 0.6 mL of 10% γ-aminobutyric acid dissolved in distilled water; TM, thermally manipulated eggs exposed to 39.6°C for 6 h daily from embryonic day 10 to 18; G10+TM, eggs that received both previous treatments during incubation. Chicks obtained were grown for 28 days.

a,b Means in a row that possess different superscripts differ significantly (p<0.05).

**Table 5:** Effects of *in ovo* feeding of γ-aminobutyric acid and embryonic thermal manipulation on absolute and relative organs weight in broiler chickens

Parameters	Treatments^1)^				SEM	p-value

	CON	G10	TM	G10+TM		
Absolute weight (g)						
Liver	36.9^ab^	41.2^a^	35.6^ab^	33.3^b^	1.1	0.046
Spleen	1.2	1.1	1.4	1.01	0.2	0.460
Bursa	1.7	2.1	1.8	2.3	0.1	0.093
Heart	7.3	8.2	6.4	6.8	0.3	0.080
Relative weight (%)						
Liver	2.9	3.3	3.1	3.1	0.08	0.264
Spleen	0.09	0.09	0.12	0.09	0.02	0.442
Bursa	0.13^B^	0.17^AB^	0.15^B^	0.22^A^	0.01	0.008
Heart	0.57	0.66	0.54	0.65	0.02	0.138

Values were obtained from n = 5 repetitions per treatment.

1) The treatments are described as follows: CON, control eggs without *in ovo* injection and incubated at standard temperature; G10, eggs injected at 17.5 days of incubation with 0.6 mL of 10% γ-aminobutyric acid dissolved in distilled water; TM, thermally manipulated eggs exposed to 39.6°C for 6 h daily from embryonic day 10 to 18; G10+TM, eggs that received both previous treatments during incubation. Chicks obtained were grown for 28 days.

a,b Means in a row that possess different superscripts differ significantly (p<0.05).

A,B Means in a row that possess different superscripts differ significantly (p<0.01).

**Table 6:** Effects of *in ovo* feeding of γ-aminobutyric acid and embryonic thermal manipulation on plasma biochemical parameters in broiler chickens

Parameters	Treatments^1)^				SEM	p-value

	CON	G10	TM	G10+TM		
Glucose (mg/dL)	251.8	274.4	274.8	266.6	6.7	0.624
Triglycerides (mg/dL)	41.4	27.6	26.6	37.6	3.2	0.267
Total protein (g/dL)	2.48^b^	2.62^ab^	2.58^ab^	3.1^a^	0.1	0.019
Cholesterol (mg/dL)	114.8	96.2	88.2	111	6.8	0.506

Values were obtained from n = 5 repetitions per treatment.

SEM, standard error of the mean.

1) The treatments are described as follows: CON, control eggs without *in ovo* injection and incubated at standard temperature; G10, eggs injected at 17.5 days of incubation with 0.6mL of 10% γ-aminobutyric acid dissolved in distilled water; TM, thermally manipulated eggs exposed to 39.6°C for 6 h daily from embryonic day 10 to 18; G10+TM, eggs that received both previous treatments during incubation. Chicks obtained were grown for 28 days.

a,b Means in a row that possess different superscripts differ significantly (p<0.05).

**Table 7:** Effects of *in ovo* feeding of γ-aminobutyric acid and embryonic thermal manipulation on relative hepatic gene expression in broiler chickens

Genes	Treatments^1)^				SEM	p-value

	CON	G10	TM	G10+TM		
*SOD*	1.00	1.58	1.28	2.36	0.33	0.522
*CAT*	1.00	1.59	3.22	4.41	0.61	0.171
*GPx1*	1.00^a^	1.31^ab^	1.59^ab^	2.04^b^	0.19	0.039
*NOX1*	1.00^B^	0.28^A^	0.52^AB^	0.43^A^	0.09	0.007
*NOX4*	1.00	0.41	0.55	0.57	0.11	0.274
*ACC*	1.00	2.26	2.17	0.41	0.52	0.573
*FAS*	1.00	1.89	1.02	0.84	0.17	0.124
*NRF2*	1.00	2.26	1.01	1.23	0.22	0.123
*EXFABP*	1.00	0.91	0.51	0.21	0.11	0.283
*PPAR-γ*	1.00	0.99	0.99	0.43	0.14	0.497

Values were obtained from n = 5 repetitions per treatment.

SEM, standard error of the mean; *SOD*, superoxide dismutase; *CAT*, catalase; *GPx1*, glutathione peroxidase 1; *NOX1*, nicotinamide adenine dinucleotide phosphate oxidase 1; *NOX4*, nicotinamide adenine dinucleotide phosphate oxidase 4; *ACC*, acetyl-CoA carboxylase; *FAS*, fatty acid synthase; *NRF2*, nuclear factor erythroid 2-related factor 2; *EXFABP*, extracellular fatty acid-binding protein; *PPAR-γ*, peroxisome proliferator-activated receptor-gamma.

1) The treatments are described as follows: CON, control eggs without *in ovo* injection and incubated at standard temperature; G10, eggs injected at 17.5 days of incubation with 0.6 mL of 10% γ-aminobutyric acid dissolved in distilled water; TM, thermally manipulated eggs exposed to 39.6°C for 6 h daily from embryonic day 10 to 18; G10+TM, eggs that received both previous treatments during incubation. Chicks obtained were grown for 28 days.

a,b Means in a row that possess different superscripts differ significantly (p<0.05).

A,B Means in a row that possess different superscripts differ significantly (p<0.01).

**Table 8:** Results of planned contrasts of hepatic gene expression analysis (p-value)

Genes	Planned contrast^1)^		

	CON vs G10	CON vs TM	CON vs G10+TM
*SOD*	NS	NS	NS
*CAT*	NS	NS	0.046
*GPx1*	NS	0.092	0.006
*NOX1*	0.001	0.019	0.007
*NOX4*	0.077	NS	NS
*ACC*	NS	NS	NS
*FAS*	0.065	NS	NS
*NRF2*	0.039	NS	NS
*EXFABP*	NS	NS	0.087
*PPAR-γ*	NS	NS	NS

1) The treatments are described as follows: CON, control eggs without *in ovo* injection and incubated at standard temperature; G10, eggs injected at 17.5 days of incubation with 0.6 mL of 10% γ-aminobutyric acid dissolved in distilled water; TM, thermally manipulated eggs exposed to 39.6°C for 6 h daily from embryonic day 10 to 18; G10+TM, eggs that received both previous treatments during incubation. Chicks obtained were grown for 28 days.

*SOD*, superoxide dismutase; NS, not significant; *CAT*, catalase; *GPx1*, glutathione peroxidase 1; *NOX1*, nicotinamide adenine dinucleotide phosphate oxidase 1; *NOX4*, nicotinamide adenine dinucleotide phosphate oxidase 4; *ACC*, acetyl-CoA carboxylase; *FAS*, fatty acid synthase; *NRF2*, nuclear factor erythroid 2-related factor 2; *EXFABP*, extracellular fatty acid-binding protein; *PPAR-γ*, peroxisome proliferator-activated receptor-gamma.
